# Transdiagnostic comparison of visual working memory capacity in bipolar disorder and schizophrenia

**DOI:** 10.1186/s40345-020-00217-x

**Published:** 2021-04-02

**Authors:** Catherine V. Barnes-Scheufler, Caroline Passow, Lara Rösler, Jutta S. Mayer, Viola Oertel, Sarah Kittel-Schneider, Silke Matura, Andreas Reif, Robert A. Bittner

**Affiliations:** 1 Department of Psychiatry, Psychosomatic Medicine, and Psychotherapy, University Hospital Frankfurt, Goethe University, Heinrich-Hoffmann-Str. 10, 60528 Frankfurt am Main, Germany; 2 Netherlands Institute for Neuroscience, Amsterdam, The Netherlands; 3 Department of Child and Adolescent Psychiatry, Psychosomatics and Psychotherapy, University Hospital Frankfurt, Goethe University, Frankfurt am Main, Germany; 4 Department of Psychiatry, Psychosomatic Medicine, and Psychotherapy, University Hospital Würzburg, Würzburg, Germany; 5 Ernst Strüngmann Institute for Neuroscience (ESI) in Cooperation with Max Planck Society, Frankfurt am Main, Germany

**Keywords:** Bipolar disorder, Schizophrenia, Cognitive dysfunction, Working memory capacity, Attention

## Abstract

**Background:**

Impaired working memory is a core cognitive deficit in both bipolar disorder and schizophrenia. Its study might yield crucial insights into the underpinnings of both disorders on the cognitive and neurophysiological level. Visual working memory capacity is a particularly promising construct for such translational studies. However, it has not yet been investigated across the full spectrum of both disorders. The aim of our study was to compare the degree of reductions of visual working memory capacity in patients with bipolar disorder (PBD) and patients with schizophrenia (PSZ) using a paradigm well established in cognitive neuroscience.

**Methods:**

62 PBD, 64 PSZ, and 70 healthy controls (HC) completed a canonical visual change detection task. Participants had to encode the color of four circles and indicate after a short delay whether the color of one of the circles had changed or not. We estimated working memory capacity using Pashler’s K.

**Results:**

Working memory capacity was significantly reduced in both PBD and PSZ compared to HC. We observed a small effect size (*r* = .202) for the difference between HC and PBD and a medium effect size (*r* = .370) for the difference between HC and PSZ. Working memory capacity in PSZ was also significantly reduced compared to PBD with a small effect size (*r* = .201). Thus, PBD showed an intermediate level of impairment.

**Conclusions:**

These findings provide evidence for a gradient of reduced working memory capacity in bipolar disorder and schizophrenia, with PSZ showing the strongest degree of impairment. This underscores the importance of disturbed information processing for both bipolar disorder and schizophrenia. Our results are compatible with the cognitive manifestation of a neurodevelopmental gradient affecting bipolar disorder to a lesser degree than schizophrenia. They also highlight the relevance of visual working memory capacity for the development of both behavior- and brain-based transdiagnostic biomarkers.

## Background

Cognitive impairment across a wide range of domains is a central common characteristic of both bipolar disorder and schizophrenia (Martínez-Arán et al. 2004; Kahn and Keefe 2013; Vöhringer et al. 2013; Bora and Pantelis 2015; Miskowiak et al. 2018). Consequently, both have been conceptualized as information processing disorders (Kahn and Keefe 2013; Bortolato et al. 2015). This paradigm supports the notion that transdiagnostic comparisons of crucial cognitive constructs are a central element of translational strategies to establish a psychiatric nosology based on the assessment of cognitive dimensions and the brain networks which give rise to them (Cuthbert 2014; Insel 2014). Ultimately, this should lead to the identification of neurobiologically distinct biotypes across diagnostic boundaries (Clementz et al. 2016) and the development of behavioral and brain-based biomarkers (Oertel-Knöchel et al. 2011). Furthermore, it might also facilitate a better understanding of the neurophysiological disturbances underlying impaired information processing and the development of more effective pro-cognitive interventions.

The need for transdiagnostic studies is underscored by the substantial phenomenological and pathophysiological overlap of bipolar disorder and schizophrenia (Ivleva et al. 2010; Pearlson 2015). They have the highest amount of shared heritability among neuropsychiatric disorders (Anttila et al. 2018; Lee et al. 2019). Both are also regarded to different degrees as neurodevelopmental disorders (Bortolato et al. 2015; Pearlson 2015), possibly forming a neurodevelopmental continuum (Owen and O'Donovan 2017). This implies that risk factors disturbing brain development and cognition play a larger role in schizophrenia than in bipolar disorder. Interestingly, most studies have reported a gradient of cognitive impairment with patients with schizophrenia generally more affected than patients with bipolar disorder (Goldberg 1999; Schretlen et al. 2007; Ivleva et al. 2010; Lewandowski et al. 2011; Hill et al. 2013; Reilly and Sweeney 2014).

Working memory is universally regarded as a central cognitive domain for transdiagnostic studies of impaired information processing (Insel et al. 2010). It is a crucial determinant of essential cognitive functions such as language comprehension and reasoning (Baddeley 1992), as well as an important mediator of cognitive development and learning (Baddeley and Hitch 1974; Cowan 2014). Working memory dysfunction is a central cognitive deficit in both bipolar disorder and schizophrenia (Glahn et al. 2006; Barch and Smith 2008). It has been reported in a large number of behavioral studies in schizophrenia across all stages of illness (Lee and Park 2005; Barch and Smith 2008; Luck and Gold 2008; Fuller et al. 2009; Hahn et al. 2010; Anticevic et al. 2011b; Leonard et al. 2017; Mayer et al. 2018). Working memory impairment has also been demonstrated in bipolar disorder (Adler et al. 2004; Glahn et al. 2006; Thompson et al. 2007; Mayer and Park 2012; Jensen et al. 2016). While working memory deficits appear to be particularly pronounced in manic or depressive phases (Townsend et al. 2010), they persist during euthymic phases of the illness (Xu et al. 2012), at least in a sizable number of patients (Volkert et al. 2015). Direct comparisons between patients with bipolar I (BP-I) and bipolar II (BP-II) disorder indicate overall a similar degree of working memory impairment (Bora et al. 2011; Bora 2018). Additionally, there is evidence for a modestly greater degree of impairment in bipolar patients with a history of psychosis, compared to bipolar patients without a history of psychosis (Bora 2018).

One particularly relevant aspect of working memory is its limited capacity (Cowan 2001), which appears to constitute a core cognitive trait with high intra-individual stability over time (Kane and Engle 2002). Working memory capacity differs considerably between individuals and has strong links to high-level cognitive measures including global fluid intelligence, abstract reasoning, language abilities, mathematics, and overall scholastic performance (Daneman and Carpenter 1980; Cowan et al. 2005; Fukuda et al. 2010; Johnson, McMahon et al. 2013; Cowan 2014; Unsworth et al. 2014). Finding pro-cognitive interventions which increase patients’ working memory capacity should therefore also be a promising way to improve their general level of cognitive functioning (Johnson et al. 2013). Quantifying the degree to which working memory capacity is constrained across the schizo-bipolar spectrum is an important step toward this goal.

Based on the extensive body of work in the field of cognitive neuroscience (Luck and Vogel 2013), visual working memory capacity has been proposed as an especially suitable construct for this purpose (Barch et al. 2012). This is supported by its good construct validity and a number of specific properties. Visual working memory capacity correlates closely with measures of verbal working memory capacity but is less prone to chunking or rehearsal mechanisms (Luck and Vogel 1997; Cowan 2001), which could confound the estimation of pure working memory capacity. It has also been studied extensively using functional neuroimaging (Linden et al. 2003; Todd and Marois 2004; Vogel and Machizawa 2004). Conversely, spatial span paradigms are generally regarded as poorly suited for functional neuroimaging studies (Barch and Smith 2008). Additionally, paradigms assessing visual working memory capacity have good test–retest reliability (Xu et al. 2018; Dai et al. 2019) and have been employed successfully in animal studies (Wright et al. 2010).

Visual working memory capacity has been studied most commonly using change detection paradigms. Here, subjects have to remember one or more features such as color, location or orientation of an array of simple visual items. Subsequently, after a short delay interval they are shown a test array and have to make a judgment, whether the test array is identical or if a single item had changed. Healthy individuals are able to store information of about four objects at one time as integrated features (Luck and Vogel 1997; Wheeler and Treisman 2002). They are able to remember three to four items when required to encode a single feature such as color, or even two features of each item such as color and location. Variations of the ‘canonical’ change detection paradigm have also been implemented (Feuerstahler et al. 2019). In change localization paradigms, subjects need to specify which item has changed. In partial-report change detection paradigms, the change decision during the test array is limited to a single item. In multiple change detection paradigms, more than one item might change during the test array.

Reduced visual working memory capacity has been observed in schizophrenia (Gold et al. 2010; Mayer et al. 2012; Hahn et al. 2018) and in bipolar disorder I with a history of psychosis (Gold et al. 2018). However, to our knowledge, no study has compared visual working memory capacity in cohorts of patients with schizophrenia and schizoaffective disorder (PSZ) and patients with bipolar disorder (PBD) representing the full spectrum of both disorders. The main goal of our study was to assess working memory capacity in PBD of all illness subtypes, as well as PSZ using a canonical change detection paradigm. We expected to observe a gradient of reduced working memory capacity with greater impairment in PSZ than in PBD.

## Methods

### Participants

We recruited 62 PBD (42 female, mean age 42.05, range: 20—61), and 64 PSZ (26 female, mean age 38.56, range: 20–57, n = 41 with schizophrenia and n = 23 with schizoaffective disorder) from psychiatric outpatient clinics in and around Frankfurt am Main, Germany. We established diagnoses of all patients according to DSM-5 criteria based on a clinical interview and careful chart review at a consensus diagnosis meeting chaired by one of the authors (R.A.B.). We pooled both patients diagnosed with schizophrenia and schizoaffective disorder because long-term diagnostic stability and inter-rater reliability of schizoaffective disorder is relatively poor (Maj et al. 2000).

The Positive and Negative Syndrome Scale (PANSS) was used to assess current psychopathology in PSZ (Kay et al. 1987). In order to establish euthymic mood state in PBD, participants were evaluated with the Young Mania Rating Scale (YMRS) and Montgomery-Åsberg Depression Rating Scale (MADRS) (Young et al. 1978; Montgomery and Åsberg 1979). Participants with YMRS values of ≥ 11 or MADRS values of ≥ 11 were excluded from our analysis.

70 matched healthy control subjects (HC), (44 female, mean age 38.61, range: 21–61) also participated. HC had no reported history of psychiatric illness, as well as no history in first-degree family members. They were recruited from the Frankfurt University campus and surrounding areas, as well as by online and printed advertisements. Current and past symptoms of psychiatric illness were ruled out using the German version of the Structural Clinical Interview SCID-I, from the Diagnostic and Statistical Manual, Version IV (Saß et al. 2003).

All participants reported no history of neurological illness and no drug use (excluding nicotine) within the past six months. All participants ranged in age from 20–61 years old. We matched subjects at the group level by conducting Kruskal–Wallis tests based on age (*H*(2) = 3.902, *p* = 0.142), and participants’ years of education (*H*(2) = 1.254, *p* = 0.534), as well as parental years of education (*H*(2) = 0.834, *p* = 0.659).

We assessed handedness as a continuous variable using the Edinburgh Handedness Inventory (Oldfield 1971). We compared handedness scores between groups using a Kruskal–Wallis test and did not find a significant difference (*H*(2) = 0.962, p = 0.618).

The German Mehrfachwahl-Wortschatz-Intelligenz Test (MWT-B) (Lehrl et al. 2005) was administered to assess premorbid verbal intelligence.

Further socio-demographic information for all cohorts can be found in Table 1. Prior to signing the informed consent form, participants were informed of its contents by the investigator and what to do in the case of experiencing distress, and how to end participation in the study. The ethics committee of the University Hospital Frankfurt approved all study procedures.

**Table 1:** Demographic and clinical characteristics of all groups

Group	n	Age (SD)	Gender	YOE (SD)	PYOE (SD)	IQ (SD)	DOI (SD)	Handed ness (SD)	YMRS (SD)	MADRS (SD)	PANSS Total (SD)	PANSS Positive (SD)	PANSS Negative (SD)
**HC**	70	38.61 (13.11)	62.86	16.27 (2.10)	14.91 (3.14)	116.03 (12.31)	–	64.50 (45.64)	–	–	–	–	–
**PBD**	62	42.05 (10.87)	67.74	16.34 (2.33)	14.67 (3.72)	117.84 (12.77)	13.57 (10.17)	71.87 (38.70)	1.66 (2.30)	3.23 (2.83)	–	–	–
BP-I	38	40.26 (10.77)	73.68	16.37 (2.03)	14.78 (3.68)	118.05 (14.50)	12.40 (9.37)	69.36 (41.86)	1.68 (2.20)	3.26 (3.00)	–	–	–
BP-II	24	44.88 (10.65)	58.33	16.29 (2.80)	14.50 (3.84)	117.50 (9.69)	15.42 (11.29)	75.83 (33.53)	1.63 (2.46)	3.17 (2.70)	–	–	–
HPS +	32	42.56 (9.60)	59.37	16.70 (1.75)	14.68 (3.74)	119.16 (13.29)	14.88 (10.34)	72.81 (34.47)	1.31 (1.87)	3.28 (2.91)	–	–	–
HPS-	30	41.50 (12.23)	76.67	15.97 (2.81)	14.67 (3.76)	116.43 (12.26)	12.17 (9.96)	70.86 (43.32)	2.03 (2.70)	3.17 (2.78)	–	–	–
**PSZ**	64	38.56 (10.30)	40.62	15.80 (2.75)	14.29 (3.73)	110.77 (13.35)	10.25 (9.22)	67.08 (44.64)	–	–	45.03 (10.35)	10.32 (3.61)	11.97 (4.21)
SZ	41	36.85 (9.57)	36.59	15.43 (2.85)	14.50 (3.78)	108.39 (11.37)	7.78 (7.41)	69.90 (38.83)	–	–	46.98 (10.53)	10.73 (4.01)	12.85 (4.34)
SZAFF	23	41.61 (11.07)	47.83	16.46 (2.48)	13.91 (3.72)	115.00 (15.69)	14.65 (10.57)	62.06 (54.08)	–	–	41.15 (9.02)	9.50 (2.54)	10.20 (3.38)
Main Group Differences	–	NS	HC > PSZ, PBD > PSZ	NS	NS	HC > PSZ, PBD > PSZ	NS	NS	–	–	–	–	–

Age reported as mean value (SD), gender as % female, YOE = mean years of education (SD), PYOE = mean value of highest reported years of education value from either parent (SD), IQ = mean value of premorbid IQ as measured by MWT-B (SD), DOI = duration of illness in years (SD), handedness as mean value measured by Edinburgh Handedness Inventory (SD); YMRS = Young Mania Rating Scale (SD), MADRS = Montgomery-Åsberg Depression Rating Scale (SD), and PANSS = Positive and Negative Syndrome Scale (SD) reported as total score and separate positive and negative subscales, NS = no significant effect

### Change detection task

**Fig. 1:**
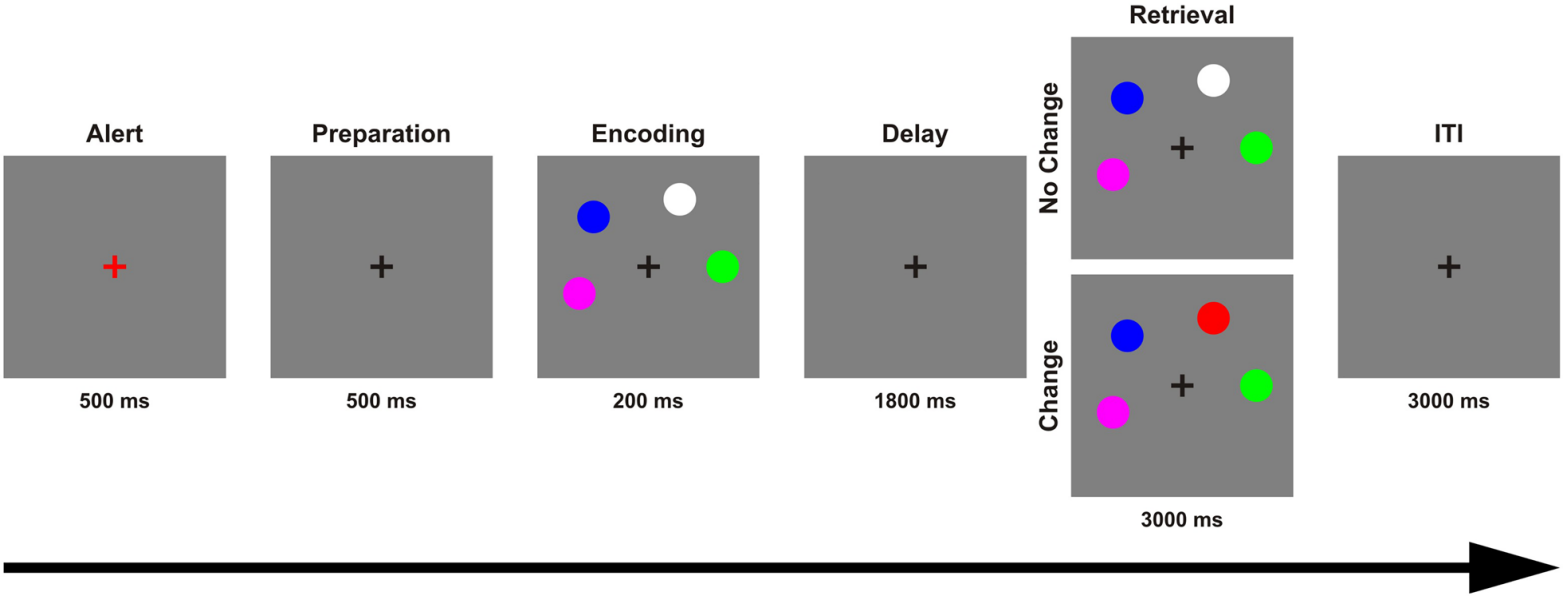
The change detection task used to assess working memory capacity. Each trial began with the alert phase, during which the fixation cross turned to red for 500 ms. This was followed by a preparation phase of 500 ms. During the encoding phase a sample array of four colored circles was presented for 200 ms. During the delay phase, the black fixation cross remained on the screen for 1800 ms. The whole-display recognition test array followed, in which participants had a maximum duration of 3000 ms to decide if the test array was identical to the sample array presented in the encoding phase, or if one of the circles had changed color

We implemented a ‘canonical’ color change detection task (Fig. 1) on a personal computer using Presentation software in Version14.9 (www.neurobs.com). Stimuli were presented on a grey background (RGB values: 191, 191, 191) in a dimly lit room with a viewing distance of approximately 60 cm. Throughout the experiment, a black fixation cross was displayed at the center of the screen. Each trial began with the alert phase, during which the fixation cross turned to red for 500 ms. This was followed by a preparation phase of 500 ms. During the encoding phase a sample array of four colored circles was presented for 200 ms. Each circle had a visual angle of approximately 0.95°. These circles were spaced equally apart on an imaginary circle with 12 possible locations around the black fixation cross covering a visual angle of approximately 5.25°, and the minimum distance between two circles was 0.29°. Each circle had one of seven easily discriminable possible colors with the following RGB values: black (0, 0, 0), red (255, 0, 0), white (255, 255, 255), blue (0, 0, 255), green (0, 255, 0), yellow (255, 255, 0), and magenta (255, 0, 255), with no repetitions of colors within a trial. During the delay phase, the black fixation cross remained on the screen for 1800 ms. A whole-display recognition test array followed, in which participants had a maximum duration of 3000 ms to decide if the test array was identical to the sample array presented in the encoding phase, or if one of the circles had changed color. Half of the trials were change trials (right mouse button), the other half no-change trials (left mouse button). In change trials, a randomly chosen circle changed its color. The total duration of each trial was 6000 ms followed by an inter-trial interval of 3000 ms. All participants received the same instructions prior to the beginning the task, and were asked to perform as accurately as possible, and to keep their eyes fixated constantly on the center of the screen. A total of 60 trials were tested in each participant, which required approximately nine minutes of testing time.

### Medication scores

Patients were on stable medication for at least one month at the time of study. One PSZ and two PBD did not receive medication. Details of medication can be found in Table 2. We calculated olanzapine equivalence scores for antipsychotic medication (Gardner et al. 2010), and daily doses for mood stabilizers (lithium, valproate and lamotrigine) in both patient groups.

**Table 2:** Medication of patients with bipolar disorder and patients with schizophrenia

Group	FGA	SGA	FGA & SGA	OES (SD)	Lithium	Lithium Dose (SD)	Valproate	Valproate Dose (SD)	Lamotrigine	Lamotrigine Dose (SD)	Anti- depressants
HC	–	–		–	–	–	–	–	–	–	–
PBD	0	35	0	5.10	29	1078.62	10	1225.00	10	185.00	23
				(6.78), 35		(401.68)		(454.15)		(57.98)	
PSZ	1	62	5	17.72	3	750.00	2	350.00	0	–	14
				(14.10),		(343.69)		(212.13)			
				62							

Number of patients receiving the different medication classes at time of study. FGA = first-generation antipsychotics, SGA = second-generation antipsychotics. Olanzapine equivalence scores (OES) calculated according to Gardener et al. (2010), reported in mean (SD), and n of patients. Lithium, valproate and lamotrigine doses reported as n of patients and mean daily mg value (SD). Antidepressants reported as n of patients receiving this type of medication

### Calculation of working memory capacity

In order to quantify the amount of information participants stored in working memory by each participant, we calculated Pashler’s *K*: *K* = N × ( (H—FA) / (1—FA) (Pashler 1988). H is the hit rate (proportion of correct responses to change trials). FA is the false alarm rate (proportion of incorrect responses to no-change trials). N is the set size, which was always four. Our whole-display recognition paradigm required the use of Pashler’s *K* as the appropriate estimate of working memory capacity (Rouder et al. 2011).We excluded datasets with accuracy at or below chance level of (50%) under the assumption that participants did not attend to the task, because they were thoroughly instructed and practiced the task with the experimenter. In total we excluded three PSZ; two had accuracies of 23%, and one 40%, one PBD with an accuracy of 23% and one HC with an accuracy of 47%.

### Comparison of working memory capacity

The goal of our primary analysis was to assess group differences of working memory capacity. A Shapiro–Wilk test across all three groups revealed that our working memory capacity estimates did not follow a normal distribution (*W*(196) = 0.88, *p* < 0.01). Consequently, we used the non-parametric Kruskal–Wallis test for our primary planned analysis in order to detect working memory capacity differences across all three groups. We then conducted Mann–Whitney U tests for pairwise comparisons and calculated the effect size with *r* = *z*/√N. We interpreted effect sizes as follows: large effect size *r* = 0.5, medium *r* = 0.3, and small *r* = 0.1 (Cohen 1988).

### Investigation of possible demographic influences

Nominally higher, but not significantly different premorbid IQ values were recorded in PBD (mean = 117.84) than HC (mean = 116.03, *r* = 0.07, *p* = 0.307). Lower premorbid IQ was recorded in PSZ (mean = 110.77) compared to PBD (*r* = 0.26, *p* < 0.001) and HC (*r* = 0.20, *p* = 0.006). Gender was also not matched across participant groups (*χ*^2^(2, 196) = 10.91, *p* = 0.004). In order to control for the possible influences of group differences in premorbid IQ and gender on working memory capacity, we performed a one-way ANCOVA with the covariates premorbid IQ and gender. In order to test possible group differences in other demographic details including age, years of education, parental years of education, and handedness, we used Kruskal–Wallis tests.

### Investigation of possible influences of psychopathology

We were also interested in the relationship between working memory capacity and clinical variables. To this end, we performed separate bivariate correlations in PBD and PSZ to examine the relationship between duration of illness, YMRS, MADRS, total PANSS scores, as well as the positive and negative subscales of PANSS with *K*.

### Investigation of possible medication effects

There is evidence for an impact of antipsychotic medication (Reilly et al. 2006) and mood stabilizers (Khan et al. 2004; Senturk et al. 2007) on cognition, which might confound our analysis. We addressed this issue as follows.

We conducted a Mann–Whitney U test in order to investigate possible group differences in olanzapine equivalence scores for both patient groups. We also conducted Spearman’s r bivariate correlations (*r*_*s*_) to examine the relationship between working memory capacity and olanzapine equivalence scores in both PSZ and PBD.

In addition, we conducted another Mann–Whitney U test to check for a possible difference between PBD receiving antipsychotic medication at the time of the study and those PBD that were not receiving antipsychotic medication. We conducted additional Spearman’s r bivariate correlations to examine the relationship between working memory capacity and daily lithium dose only in PBD because only three patients in the PSZ group received lithium treatment. Similarly, we only conducted Spearman’s r bivariate correlations between working memory capacity and daily valproate and lamotrigine doses in PBD because only two patients in the PSZ group received valproate and none received lamotrigine.

### Investigation of diagnostic subgroups

In order to evaluate possible working memory capacity differences in patient subgroups, we reviewed the pairwise comparisons of Mann–Whitney U tests of *K* in patients with (a) schizophrenia (SZ) versus schizoaffective disorder (SZAFF), (b) BP-I versus BP-II, and (c) bipolar with a history of psychotic symptoms (BP HPS +) versus bipolar without a history of psychotic symptoms (BP HPS-).

## Results

### Comparison of working memory capacity

Working memory capacity was highest in HC (Mean = 3.39, SD = 0.61) followed by PBD (Mean = 3.15, SD = 0.75) and then PSZ (Mean = 2.90, SD = 0.77). Our primary analysis revealed that *K* was significantly different across all three groups (*H*(2) = 19.43, *p* < 0.001). Pairwise group comparisons revealed a significant difference of *K* between PSZ and HC with a medium effect size (*r* = 0.370, *p* < 0.001) and between PBD and HC with a small effect size (*r* = 0.202, *p* = 0.020). There was also a significant reduction of working memory capacity in PSZ compared to PBD with a small effect size (*r* = 0.201, *p* = 0.024) (Table 3).

**Table 3:** Mean working memory (WM) capacity as estimated using Pashler’s *K* (SD)

	WM Capacity mean (SD)	Group comparisons Effect size (*p*-value)								
		HC	PBD	BP-I	BP-II	BP HPS +	BP HPS-	PSZ	SZ	SZAFF
HC	3.39 (0.61)	–	0.202 (0.020)*	0.167 (0.082)	0.214 (0.038)*	0.216 (0.029)*	0.156 (0.120)	0.370 (< 0.001)*	0.340 (< 0.001)*	0.347 (< 0.001)*
PBD	3.15 (0.75)	0.202 (0.020)*	–	–	–	–	–	0.201 (0.024)*	–	–
BP-I	3.22 (0.62)	0.167 (0.082)	–	–	0.104 (0.414)	–	–	–	0.211 (0.061)	0.290 (0.025)*
BP-II	3.03 (0.92)	0.214 (0.038)*	–	0.104 (0.414)	–	–	–	–	0.105 (0.395)	0.180 (0.217)
BP HPS +	3.19 (0.56)	0.216 (0.029)*	–	–	–	–	0.038 (0.767)	–	0.169 (0.148)	0.262 (0.052)
BP HPS-	3.10 (0.92)	0.156 (0.120)	–	–	–	0.038 (0.767)		–	0.171 (0.150)	0.233 (0.090)
PSZ	2.90 (0.77)	0.370 (< 0.001)*	0.201 (0.024)*	–	–	–	–	–	–	–
SZ	2.89 (0.81)	0.340 (< 0.001)*	–	0.211 (0.061)	0.105 (0.395)	0.169 (0.148)	0.171 (0.150)	–	–	-0.047 (0.705)
SZAFF	2.90 (0.70)	0.347 (< 0.001)*	–	0.290 (0.025)*	0.180 (0.217)	0.262 (0.052)	0.233 (0.090)	–	-0.047 (0.705)	–

Results of Mann–Whitney U tests to test pairwise comparisons of working memory capacity estimated with Pashler’s K and reported as effect size (*r* = *z* / √N) and *p*-value. HC = healthy controls, PBD = patients with bipolar disorder, BP-I = patients with bipolar type I, BP-II = bipolar patients with bipolar type II, BP HPS +  = bipolar patients with history of psychotic symptoms, BP HPS- = bipolar patients without history of psychotic symptoms, PSZ = patients with schizophrenia, SZ = patients with schizophrenia, SZAFF = patients with schizoaffective disorder. Statistical significance is indicated by *

### Investigation of possible demographic influences

In a one-way ANCOVA there was no significant relation of premorbid IQ, the covariate, to *K* across all groups (*F*(1, 191) = 0.19, partial *Ƞ*^2^ < 0.01, *p* = 0.661). There was also no significant relation of gender, the covariate to *K* across all groups (*F*(1, 191) = 1.56, partial *Ƞ*^2^ < 0.01, *p* = 0.213). A significant effect of group on *K* was still observed after controlling for both the effects of premorbid IQ and gender (*F*(2, 191) = 6.90, partial *Ƞ*^2^ = 0.07, *p* = 0.001).

### Investigation of possible psychopathological influences

There was no significant correlation between *K* and years of illness in either PBD (*r*_*s*_ = –0.15, *p* = 0.254, *n* = 62), or PSZ (*r*_*s*_ < –0.01, *p* = 0.961, *n* = 64). There was no significant correlation between *K* and scores on the YMRS (*r*_*s*_ = 0.10, *p* = 0.449), or MADRS (*r*_*s*_ = –0.15, *p* = 0.239) in PBD. There was no significant correlation between *K* and total PANSS scores (*r*_*s*_ = –0.15, *p* = 0.242), as well as PANSS positive (*r*_*s*_ = –0.22, *p* = 0.093), and PANSS negative subscale (*r*_*s*_ < 0.01, *p* = 0.947) in PSZ.

### Investigation of possible medication effects

We recorded higher olanzapine equivalence scores in PSZ (*Mdn* = 13.41) compared to PBD (*Mdn* = 2.50) (*U* = 626.50, *z* = –6.60, *r* = 0.67, *p* < 0.001). Yet, we observed no significant correlation of *K* with olanzapine equivalence scores in either PSZ (*r*_*s*_ = –0.16, *p* = 0.202, *n* = 62), or PBD (*r*_*s*_ = –0.14, *p* = 0.285, *n* = 35). No significant difference was detected between *K* in PBD taking antipsychotic medication (*Mdn* = 3.31) and those not taking antipsychotic medication (*Mdn* = 3.41) (*U* = 427.00, *z* = –0.646, *r* = 0.08, *p* = 0.518). Similarly, we observed no significant correlation between *K* and daily lithium dose in PBD (*r*_*s*_ = –0.07, *p* = 0.721, *n* = 29), as well as *K* and daily valproate dose (*r*_*s*_ = –0.39, *p* = 0.263, *n* = 10), and *K* and daily lamotrigine dose (*r*_*s*_ = –0.51, *p* = 0.136, *n* = 10) in PBD.

### Investigation of diagnostic subgroups

Regarding our exploratory patient subgroup analyses, we observed no significant differences of *K* in any of the following Mann–Whitney U tests. This included the comparison of (a) schizophrenia (SZ) versus schizoaffective disorder (SZAFF) (*r* = 0.047, *p* = 0.705), (b) bipolar I (BP-I) versus bipolar II (BP-II) (*r* = 0.104, *p* = 0.414), and (c) bipolar with a history of psychotic symptoms (BP HPS +) versus bipolar without a history of psychotic symptoms (BP HPS-) (*r* = 0.038, *p* = 0.767) (Table 3). We consider these analyses exploratory as the sample sizes were relatively small, groups were not matched well and the effect sizes were all small.

## Discussion

We investigated working memory capacity in bipolar disorder and schizophrenia compared to healthy controls in order to elucidate the degree of working memory impairment in these major psychiatric disorders. We observed a significant reduction in working memory capacity in PBD compared to HC and in PSZ compared to HC. Working memory capacity of PBD fell between PSZ and HC with a significant difference between both patient groups.

Our results indicate a gradient of reduced working memory capacity across the schizo-bipolar spectrum, with PSZ showing a stronger impairment with a medium effect size. By comparison, working memory capacity reduction in PBD was less pronounced with a small effect size. These findings match previous studies on working memory dysfunction in both disorders, which indicate a similar intermediate level of working memory impairment in PBD (Hamilton et al. 2009; Barch and Sheffield 2014). Our findings are also well in line with previous studies demonstrating a comparable gradient of impairment across a wide range of other cognitive domains (Goldberg 1999; Schretlen et al. 2007; Ivleva et al. 2010; Lewandowski et al. 2011; Hill et al. 2013; Reilly and Sweeney 2014).

Our three participant groups differed in their levels of premorbid IQ. We report significantly higher premorbid IQ in PBD and HC as compared to in PSZ, and no significant difference between PBD and HC. However, there is evidence, that premorbid intelligence scores tend to be lower in PSZ (Crawford et al. 1992; Khandaker et al. 2011), and supranormal in multiple measurements in PBD (Bora 2015). Nevertheless, we did not observe a significant influence of premorbid IQ on *K*, which is unsurprising considering we measured crystallized intelligence. In comparison, fluid intelligence seems to be correlated with working memory capacity (Fukuda et al. 2010).

Recently, a large multi-center study using both a change localization and a multiple change detection task reported an overall reduction of visual working memory capacity across psychotic disorders, i.e. schizophrenia, schizoaffective disorder and BP-I with a history of psychosis (Gold et al. 2018). The authors did not observe a significant difference between any of the three patient groups in either task. For the change localization task, visual working memory capacity was significantly lower in all three patient groups. Interestingly, for the multiple change detection task this reduction was most pronounced in patients with bipolar I disorder with a lifetime history of psychosis, with only a trend towards a significant reduction in patients with schizophrenia. These results indicate that visual working memory capacity reduction in BP-I with a lifetime history of psychosis is similar in magnitude to schizophrenia. However, it remains unclear, whether this similarity is attributable to the shared presence of psychosis, because the study did not include either BP-I without a lifetime history of psychosis or BP-II. In contrast to Gold et al., we observed an intermediate level of visual working memory capacity reduction in PBD. Several factors could account for this discrepancy. Firstly, we studied the full bipolar spectrum including BP-I without a history of psychosis and BP-II, rather than focusing only on the effect of psychotic illness across diagnostic categories. Interestingly, our exploratory subgroup analyses did not indicate a difference in visual working memory capacity between BP-I and BP-II. We also did not observe an effect of lifetime history of psychosis within the PBD group. However, these post-hoc findings need to be interpreted with caution due to the relatively small size of our subgroups.

**Fig. 2:**
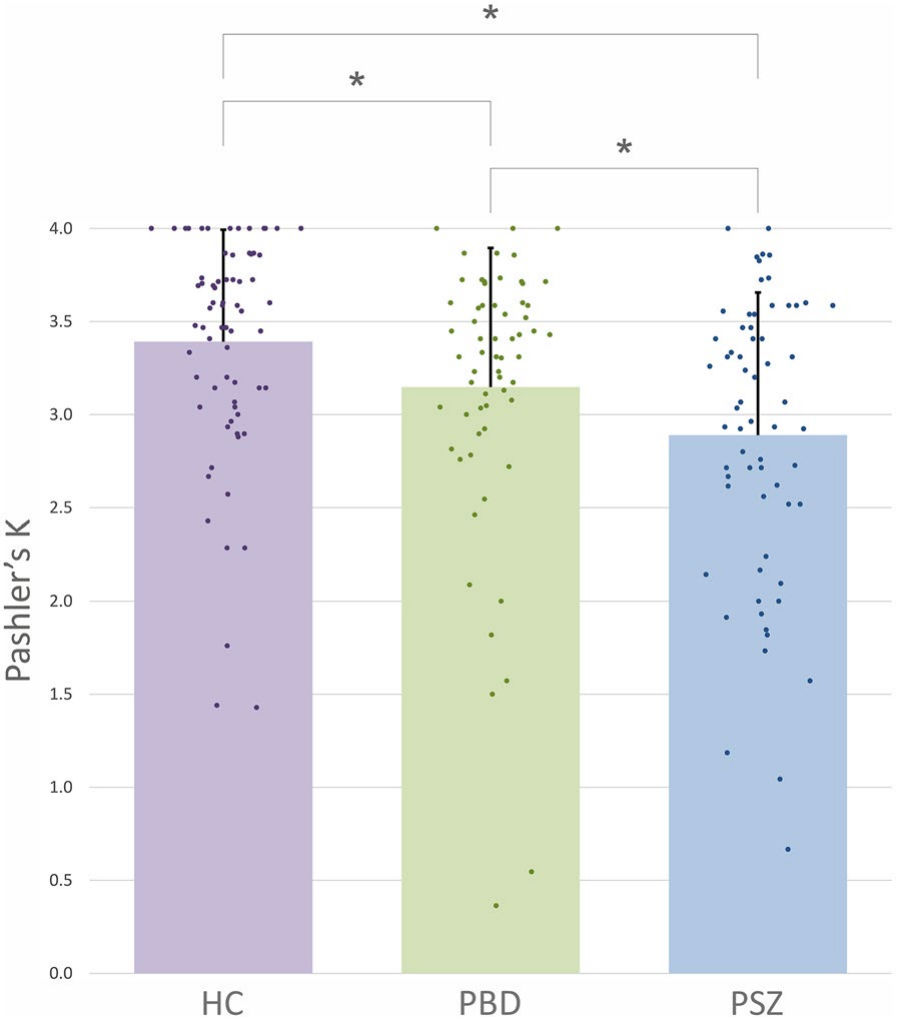
Working memory capacity for all groups. Working memory capacity was estimated using Pashler’s *K*. Bars indicate mean group average and scatter plot data indicates individual capacity estimates for healthy controls (HC) in purple, patients with bipolar disorder (PBD) in green, and patients with schizophrenia (PSZ) in blue. Error bars indicate standard deviation

There were also important differences in the paradigms employed. The number of possible changes might have influenced our results. Gold et al. observed a significant difference between healthy controls and all patient groups for their change localization task, which was a single change paradigm. By contrast, group differences were less pronounced in their multiple change detection paradigm with either zero, one, two, or five changes. Furthermore, we consistently maintained a set size of four objects, while Gold et al. used a set size of five objects. This difference might have affected results, because performance continues to decline as set size increases (Luck and Vogel 1997). This interpretation would also be in line with the notion, that deficits in PBD become more pronounced in tasks greatly exceeding their working memory capacity. It has been suggested, that a larger set size might have influenced subjects’ strategies and minimized between-group differences (Gold et al. 2018). Conversely, there was some indication of a ceiling effect in the HC group in our data with 13 HC having a *K* of 4.0 compared to three PBD and two PSZ (Fig. 2). This implies, that our set size might have underestimated working memory capacity differences between HC and both PBD and PSZ. Using both set sizes within the same study might help to clarify this issue.

Our observation of a transdiagnostic reduction of working memory capacity across the schizo-bipolar spectrum raises the question, which cognitive mechanisms might be responsible. It has been proposed that the limits of working memory capacity are determined primarily by the amount of information which can be held in the focus of attention (Cowan 2001). Specifically, top-down attention appears to be crucial for the selection of information to be stored in working memory. The efficiency of this “gatekeeper” function has a substantial impact on working memory capacity (Vogel et al. 2005; Cowan and Morey 2006; McNab and Klingberg 2008). Thus, impaired attentional processes in patients might have contributed to our results. To our best knowledge, the potential impact of attentional dysfunction on working memory impairment in bipolar disorder remains unknown. However, for schizophrenia there is experimental support for the presence of a selective impairment of top-down attentional control, which may disturb working memory encoding (Mayer et al. 2012). Furthermore, there is considerable evidence for a tendency in PSZ to show hyperfocusing of attention when processing visual information (Luck et al. 2019b). In the context of visual working memory, hyperfocusing might limit the amount of items which PSZ can focus on, thereby restricting the amount of information they can successfully encode (Leonard et al. 2013; Luck et al. 2019a).

This interpretation would also be compatible with a component-process model of working memory dysfunction in PSZ, which is based on converging behavioral and neuroimaging evidence for a primary impairment of working memory during the encoding stage (Tek et al. 2002; Hartman et al. 2003; Lee and Park 2005; Kim et al. 2006; Javitt et al. 2007; Fuller et al. 2009; Gold et al. 2010; Hahn et al. 2010; Anticevic et al. 2011a; Mayer et al. 2012).

Deficits during working memory encoding have been linked to impaired early-stage visual processing (Haenschel et al. 2007, 2009) as well as to disturbed interactions between prefrontal areas and visual areas closely involved in object processing (Bittner et al. 2015). So far it remains unclear, to which degree disturbances during the initial encoding of information contribute specifically to reduced working memory capacity in bipolar disorder and schizophrenia. Interestingly, there is evidence for differential mechanisms underlying working memory impairment in schizophrenia and bipolar disorder. While both groups were impaired in a spatial delayed response task, only patients with schizophrenia recorded more false memory responses by confidently responding that the information was correctly encoded even though it was not (Mayer and Park 2012).

Furthermore, there is evidence for an additional impairment of working memory maintenance in schizophrenia (Reilly et al. 2006; Stephane and Pellizzer 2007; Badcock et al. 2008), which could also contribute to reduced working memory capacity. Conversely, so far the presence of working memory maintenance deficits has not been investigated in patients with bipolar disorder. Future studies should try to elucidate the contribution of specific component processes to reduced working memory capacity across both disorders.

Importantly, two different models for working memory storage have been discussed. Originally, a discrete-slots model was proposed, where a specific number of items are stored up to capacity, and nothing is stored from the remaining items (Miller 1956; Luck and Vogel 1997; Cowan 2001). More recently, working memory capacity has been studied using a limited-resource model in which a dynamic precision resource spreads out across objects, such that a smaller amount of objects are encoded with higher precision (Bays and Husain 2008; Peters et al. 2018). While our paradigm was able to measure working memory capacity in a slot model, both the discrete-slot model and limited-resource model should be studied within the same patient groups.

It remains to be seen, whether the gradient of working memory capacity reduction is attributable to a comparable gradual manifestation of the same neurophysiological disturbances across diagnostic categories. Our findings also need to be reconciled with the disconnection hypothesis (Friston and Frith 1995), a parsimonious and well validated model for core cognitive and clinical features of schizophrenia that has also been recently applied to bipolar disorder (Perry et al. 2019). Functional neuroimaging studies are required to illuminate these issues.

## Conclusion

To summarize, our data provide evidence for reduced visual working memory capacity in both bipolar disorder and schizophrenia. The observed gradient of cognitive dysfunction is compatible with the neurodevelopmental continuum model (Owen and O'Donovan 2017), which would place bipolar disorder between schizophrenia and healthy controls on a neurodevelopmental gradient, matching the degree and persistence of overall functional impairment in both disorders. This interpretation should be validated by investigating the transdiagnostic impact of genetic risk variants – especially CNVs – in genetic pathways regulating neurodevelopment on working memory capacity. However, there is also evidence for a contribution of additional pathophysiological factors such as inflammation (Bora 2019; Millett et al. 2019) to cognitive impairment in both disorders. Future studies should try to determine the specific contributions of such factors to reduced visual working memory capacity. Similarly, it remains an open question how shared and distinct genetic and environmental risk factors for either disorder might influence working memory capacity on the cognitive and neurophysiological level. Given their relevance for patients’ functional capacity, future studies should also examine whether pro-cognitive interventions such as cognitive remediation could improve these deficits across diagnostic categories. Finally, our results highlight the utility of established constructs based on cognitive neuroscience for the investigation of impaired information processing in bipolar disorder similar to such endeavors in schizophrenia research (Barch and Smith 2008).

## Data Availability

The datasets used during the current study are available from the corresponding author on reasonable request.

## Abbreviations

PBD: Patients with bipolar disorder
PSZ: Patients with schizophrenia
HC: Healthy controls
YMRS: Young Mania Rating Scale
MADRS: Montgomery-Åsberg Depression Rating Scale
PANSS: The Positive and Negative Syndrome Scale
BP-I: Patients with bipolar I
BP-II: Patients with bipolar II
SZ: Patients with schizophrenia
SZAFF: Patients with schizoaffective disorder
BP HPS +: Bipolar with a history of psychotic symptoms
BP HPS-: Bipolar without a history of psychotic symptoms
*K*: Pashler’s K
SCID-I: Structural Clinical Interview
SD: Standard deviation
YOE: Years of education
PYOE: Parental years of education
DOI: Duration of illness
MWT-B: German Mehrfachwahl-Wortschatz-Intelligenz Test
